# Sex differences in inappropriate imaging requests: insights from the Medical Imaging Decision And Support (MIDAS) study

**DOI:** 10.1007/s00330-025-12088-w

**Published:** 2025-11-06

**Authors:** Stijntje Willemijn Dijk, Claudia Wollny, Thomas Kroencke, M. G. Myriam Hunink

**Affiliations:** 1 Department of Radiology and Nuclear Medicine, Erasmus MC University Medical Center, Rotterdam, The Netherlands; 2 Department of Epidemiology and Biostatistics, Erasmus MC University Medical Center, Rotterdam, The Netherlands; 3 Department of Radiology, Elisabeth-Tweesteden Ziekenhuis, Tilburg, The Netherlands; 4 Department of Diagnostic and Interventional Radiology, University Hospital Augsburg, Augsburg, Germany; 5 Centre for Advanced Analytics and Predictive Sciences (CAAPS), University of Augsburg, Augsburg, Germany; 6 Centre for Health Decision Science, Harvard T.H. Chan School of Public Health, Boston, MA United States of America

**Keywords:** Sex differences, Diagnostic imaging, Clinical decision support systems, Guidelines as topic, Healthcare costs

## Abstract

**Objective:**

Inappropriate diagnostic imaging can lead to unnecessary radiation exposure, delayed diagnoses, and increased healthcare costs. While multiple factors contribute to inappropriate imaging, sex-based disparities remain understudied. This study investigates whether inappropriate imaging requests differ among women and men.

**Materials and methods:**

We analyzed baseline data from the MIDAS study, a multi-center cluster-randomized trial conducted in three academic hospitals in Germany. Imaging requests submitted via computerized physician order entry systems were evaluated for appropriateness using the ESR iGuide, a clinical decision support tool based on the American College of Radiology Appropriateness Criteria. Requests were classified as appropriate, conditionally appropriate, or inappropriate. We compared the proportion of inappropriate requests between male and female patients using chi-square tests and calculated odds ratios, applying Bonferroni corrections for multiple comparisons.

**Results:**

Among 61,220 scored imaging requests, 31,025 were for women and 30,195 for men. The proportion of inappropriate requests was significantly higher in women (7.32%) compared to men (6.08%) (OR 1.22, 99% CI: 1.12–1.33, *p* < 0.001), with significant differences observed in subgroups for MR and among patients aged 25–65.

**Conclusion:**

Women were more likely than men to receive inappropriate imaging requests, suggesting potential disparities in diagnostic decision-making. Addressing these gaps will require further research and more sex- and gender-sensitive approaches in clinical decision-making and guideline development to ensure equitable imaging practices.

**Key Points:**

***Question*** *Inappropriate diagnostic imaging incurs radiation exposure and costs. Sex-based disparities in this context are understudied; do inappropriate imaging requests differ between men and women?*

***Findings*** *Among 61,220 requests, inappropriate orders were significantly higher in women (7.32%) than in men (6.08%).*

***Clinical relevance*** *Our results suggest sex differences in diagnostic decision-making. Further research and sex- and gender-sensitive approaches in guideline development are needed to ensure equitable imaging practices.*

**Graphical Abstract:**

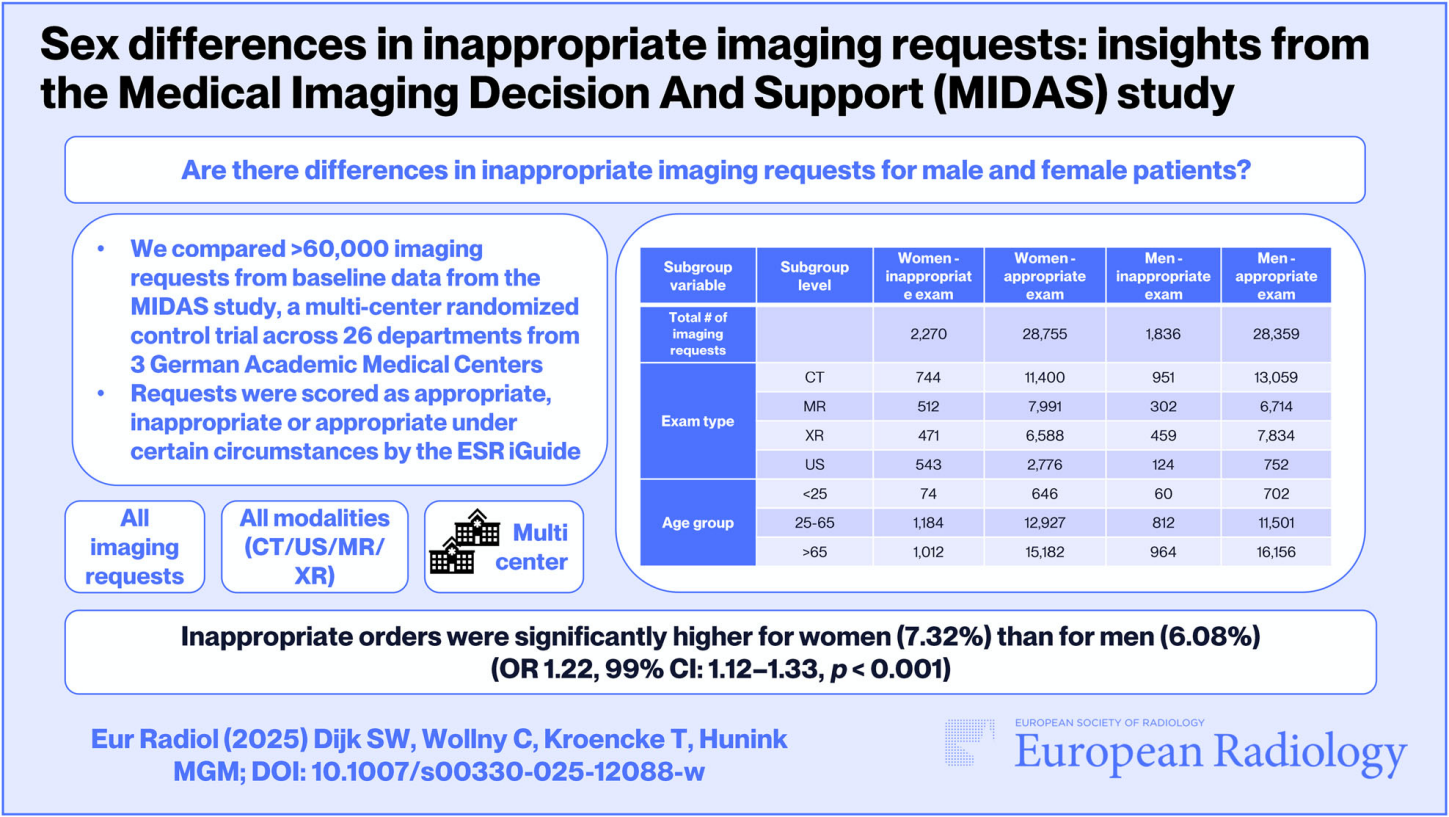

## Introduction

Diagnostic imaging is essential for accurate diagnostic and treatment decisions. However, inappropriate imaging requests are a growing concern due to overutilization, unnecessary radiation exposure, delayed diagnoses, and increased healthcare costs [1–4]. Given the widespread use of diagnostic imaging, all factors relating to imaging appropriateness should be explored.

Multiple factors contribute to inappropriate imaging, including clinical uncertainty, time pressure, medico-legal concerns, patient expectations, and variation in physician knowledge or adherence to guidelines. As health systems strive to improve the value and efficiency of care, understanding these drivers is essential. One such factor that may play a role, but has received limited attention, is the sex of the patient.

Sex differences in healthcare delivery and outcomes are well documented. Women and men often present with different symptoms, receive different diagnostic evaluations, and may experience disparities in treatment and care quality. In the context of diagnostic imaging, emerging evidence suggests sex-based differences in utilization patterns. For example, studies have shown that women may be less likely to receive appropriate imaging in emergency settings or for cardiac evaluation, while more frequently undergoing certain tests, such as venous ultrasonography for suspected deep vein thrombosis, without clear differences in clinical risk [5–8]. Women also undergo diagnostic imaging more frequently for lower back pain, whereas men are more often recommended surgical intervention [9]. Similarly, women with ischemic stroke experience greater emergency room delays than men that cannot be explained by their presenting symptoms, time of arrival, age, or other confounders [10]. Physicians may also assume somatization more often in women, offering fewer interventions [11, 12].

These findings point to potential disparities in imaging appropriateness, which could reflect unconscious biases, guideline gaps, or systemic differences in how clinical decision-making is applied across sexes.

Despite these findings, a comprehensive assessment of sex-based disparities in imaging appropriateness across modalities, age groups, and clinical settings remains lacking. Most prior studies have focused on specific imaging types or conditions and have not systematically evaluated whether women and men are equally likely to receive imaging that aligns with clinical guidelines. A recent study by Singer et al, examining CT scan appropriateness using the ESR iGuide, revealed varying results across different countries. In several countries, appropriateness was found to be lower in females compared to males—including in Belgium (71% vs. 81%), Estonia (65% vs. 72%), Greece (55% vs. 60%), and Slovenia (77% vs. 91%). Conversely, some countries showed higher appropriateness rates for females, including Denmark (88% vs. 84%), Finland (80% vs. 78%), and Hungary (77% vs. 74%) [13]. Other investigations on imaging appropriateness using the iGuide did not provide sex-specific information on inappropriateness [13–18].

To address this gap, the present study investigates sex-related differences in the appropriateness of imaging requests using data from the Medical Imaging Decision and Support (MIDAS) trial—a large, multi-center evaluation of diagnostic imaging practices in German academic hospitals. By leveraging the ESR iGuide, a validated decision support system based on the American College of Radiology Appropriateness Criteria, we systematically assess whether women and men differ in the likelihood of receiving imaging deemed inappropriate [19, 20].

## Materials and methods

This study analyzed baseline data from the Medical Imaging Decision and Support (MIDAS) trial, a multicenter, cluster-randomized controlled trial conducted across 26 departments from three German academic hospitals [19–23]. The MIDAS trial investigated the impact of a clinical decision support system (CDSS) on the appropriateness of diagnostic imaging requests in routine clinical practice. Approval from the relevant Medical Research Ethics Committees was obtained under protocol numbers 20-069 (Augsburg), B 238/21 (Kiel), 20-318 (Lübeck), and 2020-15125 (Mainz), and the trial was registered on ClinicalTrials.gov (NCT05490290).

The present analysis focuses on all imaging requests made during the pre-intervention (blinded) phase of the trial, representing a 15-month baseline period (December 2021–February 2023). Requests were submitted via the hospitals’ Computerized Physician Order Entry (CPOE) systems and included structured indication input. All imaging modalities were included in our study, which analyzed requests for computed tomography, magnetic resonance imaging, X-ray and ultrasound. Consequently, if a physician or department conducted an ultrasound independently—such point-of-care abdominal ultrasound—without submitting a formal CPOE request to the radiology department, these exams were not captured in our dataset. All requests were evaluated for appropriateness using the ESR iGuide, a CDSS based on the American College of Radiology Appropriateness Criteria and adapted for European use [24].

Each imaging request was scored automatically by the iGuide, assigning one of three categories: “appropriate” (green), “appropriate under certain conditions” (yellow), or “inappropriate” (red). A request was classified as inappropriate if the selected imaging modality was unlikely to be indicated given the clinical scenario or if the expected risk-benefit ratio was considered unfavorable. Requests that could not be scored due to unmatched indication-exam combinations or non-standard inputs were excluded from the appropriateness analysis. For example, if a physician selected an indication such as “pain left foot” but for that indication requested an “MRI of the right arm,” the system would not be able to assign a score, as no valid score for such a combination exists within the iGuide’s system. In cases where multiple indications were selected, the highest appropriateness score was used. Physicians were blinded to the appropriateness scores during the baseline phase. This choice in design aims to capture unbiased patterns of imaging behavior without any active decision support influencing their choices. As such, our results reflect current clinical practice prior to the provision of active decision support.

For this analysis, we investigated the prevalence of sex-based differences in the appropriateness of imaging requests. The analysis included only those imaging requests for which a valid appropriateness score was available and where the patient’s sex was recorded as male or female. Requests with unknown sex (*n* = 23) were excluded. The sex recorded in the hospital’s administrative system reflects the binary classification used during clinical registration and does not include gender-diverse categories.

The primary outcome of this analysis was the proportion of inappropriate imaging requests among women and men. We used chi-square (*χ*^2^) tests to compare the proportion of inappropriate requests between sexes, applying a conservative significance threshold of α = 0.01 as specified in the MIDAS protocol, and used a Welch two-sample T-test to compare the mean ages between unscored and scored requests [19]. Odds ratios (ORs) and 99% confidence intervals (CIs) were calculated to quantify the strength of associations. Secondary analyses examined the prevalence of sex differences across age groups and imaging modalities. To control for multiple comparisons, Bonferroni corrections were applied. All statistical analyses were conducted using R statistical software [25].

## Results

**Fig. 1:**
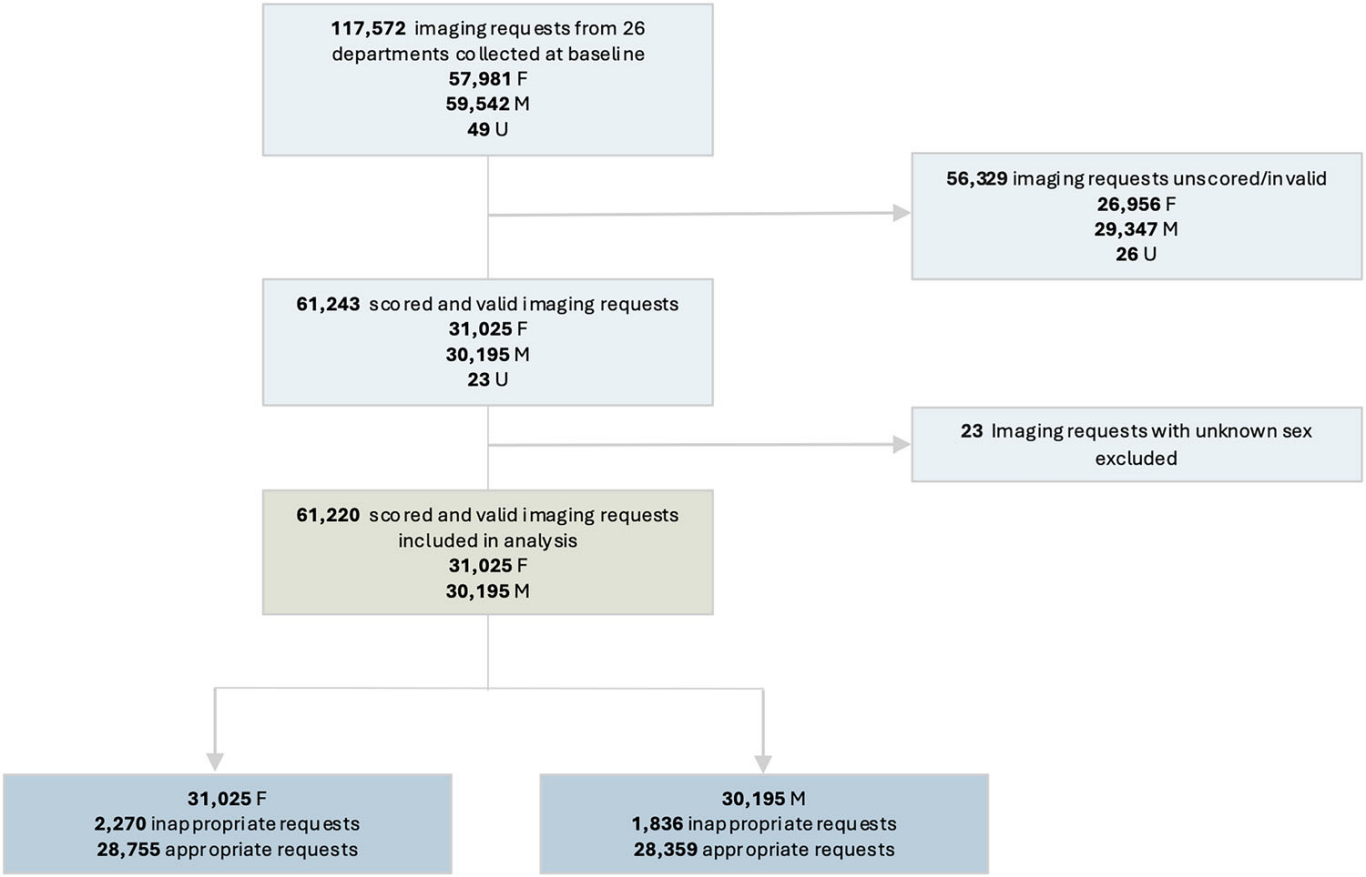
Study flowchart. F, female; M, male; U, unknown

Of 117,572 imaging exams requested from participating departments, 61,243 (52.09%) received a valid score. Unscored requests had no combination of indication and exam type that linked to a recommendation by the iGuide (*n* = 56,329). The percentage of women was higher among unscored requests (47.9% vs. 50.7%, *p* < 0.001), and the age was lower (mean age 63 vs. 61, *p* < 0.001) (Fig. 1).

Among scored requests, 23 requests were excluded as sex was unknown. The final analysis included 30,195 exams for men and 31,025 for women (Table 1).

**Table 1:** Cross-tabulation containing the original summary-level data of inappropriate exam frequency by sex, modality and age group

Subgroup variable	Subgroup level	Women—inappropriate exam	Women—appropriate exam	Men—inappropriate exam	Men—appropriate exam
Total no. of imaging requests	Total	2270	28,755	1836	28,359
Exam type	CT	744	11,400	951	13,059
	MR	512	7991	302	6714
	XR	471	6588	459	7834
	US	543	2776	124	752
Age group	< 25	74	646	60	702
	25–65	1184	12,927	812	11,501
	> 65	1012	15,182	964	16,156

The table displays the number of exams conducted across various imaging modalities (CT, MR, XR, US) and stratified by age group (< 25, 25–65, > 65)

*CT* computed tomography, *MR* magnetic resonance imaging, *XR* X-ray, *US* ultrasound

Overall, 6.70% of requests were judged as inappropriate. The proportion of inappropriate requests was higher in women (7.32%) than in men (6.08%; *χ*^2^ = 37.176, *p* < 0.001, OR 1.22 [99% CI 1.12–1.33]). This difference was also present in the subgroups for the ages 25–65 and MRs (*p* < 0.001) (Table 2).

**Table 2:** Percentage of inappropriate imaging requests in men and women, stratified by age group and exam modality (CT, MR, XR, US)

Subgroup variable	Subgroup level	% Inappropriate exam (women)	% Inappropriate exam (men)	*p*-value		OR (women vs. men) [99% CI]
Overall study	Overall	↑7.32%	6.08%	< 0.001*		1.22 [1.12–1.33]
Exam type	CT	6.13	↑6.79	0.030		0.90 [0.79–1.02]
	MR	↑6.02	4.3	< 0.001*		1.42 [1.18–1.73]
	XR	↑6.67	5.53	0.003		1.22 [1.02–1.45]
	US	↑16.36	14.16	0.112		1.19 [0.90–1.57]
Age group	< 25	↑10.28	7.87	0.107		1.34 [0.84–2.14]
	25–65	↑8.39	6.59	< 0.001*		1.30 [1.15–1.47]
	> 65	↑6.25	5.63	0.017		1.12 [0.99–1.26]

Asterisks indicate statistically significant differences at the adjusted alpha level of 0.0014 (*p* = 0.01/7). ↑ indicates the percentage of inappropriateness is highest for this sex

*CT* computed tomography, *MR* magnetic resonance imaging, *XR* X-ray, *US* ultrasound, *OR* odds ratio of inappropriate requests in women compared to men

## Discussion

In our study, the odds were 22% higher for clinicians to request inappropriate imaging for women. While the absolute percentage differences were small (1.24%), the disparity warrants further investigation. In the MR and 25–65-year age group, women also had a significantly higher proportion of inappropriate imaging requests. While not statistically significant at our stringent alpha level, all remaining age groups and modalities, except for CT, showed higher proportions of inappropriate requests for women.

Little previous research has been done on the sex-specific appropriateness of imaging requests. The recent study by Singer et al found mixed results with several countries finding more inappropriateness on CT ordering among women (Belgium, Estonia, Greece, and Slovenia) and higher appropriateness in Denmark, Finland and Hungary [13, 14, 26]. Our (German) study CT subgroup showed no statistically significant difference, though appropriateness was higher among women. The authors, like us, suggest that further research would be required to better understand the origin of these discrepancies.

The higher prevalence of inappropriate requests in women may be caused by a variety of factors, both human- and technology-driven. It may reflect differences in clinician awareness and adherence to imaging guidelines when ordering for women due to, including unconscious biases or lack of sex-specific training in diagnostics. Previous research suggests that sex and gender stereotypes can influence clinical judgment, potentially affecting both treatment and diagnostic decisions [27]. For example, clinicians may perceive older female patients as more frail or vulnerable, leading to more cautious or protective decisions that are not always aligned with guidelines or patient preferences [11, 12, 27]. In the context of imaging, such biases could contribute to deviations from guideline-based appropriateness, especially if clinicians apply different thresholds for ordering or withholding exams based on patient sex. This highlights the need to consider not only guideline adherence but also the potential impact of implicit attitudes in clinical training and support systems.

Decision support tools, when well-integrated, might help reduce these discrepancies by standardizing imaging decisions and prompting more consistent application of clinical criteria. However, the main results of the MIDAS trial did not find a reduction in inappropriate imaging requests after decision support was introduced [20, 22]. Alternatively, the imaging guidelines may not fully capture female-specific considerations in appropriateness, leading to clinically justified requests being classified as inappropriate.

The ESR iGuide has been developed with the intention of considering factors such as age and sex, and does include some female-specific recommendations [24]. Nevertheless, research underlying these guidelines is likely, as in much of medicine, to be predominantly based on studies involving men [28, 29]. This reflects a historical trend in which treatment plans and medical guidelines have often been developed primarily using male data. While not specific to imaging, a recent review of the representation of female research participants in the development of national clinical guidelines compared to the proportion of women affected by these conditions, for example, found a significant underrepresentation of women in research on antiarrhythmics, though also a slight overrepresentation in some women, such as colorectal cancer and chronic fatigue studies [30].

Our study evaluates whether imaging requests align with the iGuide’s recommendations, which are applied based on the available clinical information. As such, our findings reflect adherence to these established guidelines rather than providing direct insight into potential bias within the guidelines themselves. It is plausible that physicians may make decisions that deviate from guideline recommendations based on more nuanced or recent clinical knowledge. In such cases, particularly where female-specific considerations may not be fully captured by guidelines, a request might be rated as inappropriate by the iGuide even if it is clinically justifiable. This underscores an important area for further research in refining and updating guidelines to reflect a more inclusive evidence base. Guideline developers should consider the use of sex-disaggregated data and critically assess the applicability of evidence to all patient populations, ensuring that reference populations used to inform recommendations accurately reflect those most affected by the condition in question.

We did not have sufficient data available to reassess individual justifications for exams that were deemed inappropriate by the CDSS. While our descriptive analysis did not examine the underlying causes of the identified disparities, the findings underscore the need for further investigation and heightened awareness among both requesting clinicians and radiologists when considering imaging for women.

This study’s strength lies in the real-world integration of the ESR iGuide within routine hospital workflows, allowing us to assess imaging appropriateness in a clinical setting across a wide range of departments, exam types, and clinical indications. By embedding the decision support tool into the computerized physician order entry system without alerting physicians to appropriateness scores at baseline, we were able to capture unbiased patterns of imaging behavior.

However, several limitations warrant consideration. First and foremost, despite the iGuide’s extensive coverage of 15,000 Appropriate Use Criteria across all modalities, many requests (48%) remained unscored. This represents a considerable proportion and highlights a limitation of the clinical decision support system used. It remains unclear whether this is primarily due to limited scenario coverage in the iGuide, variability in how clinicians select indication-modality pairs, or other workflow-related issues. Potentially, this large percentage can stem from, on the one hand, clinical scenarios for which no valid combination between indication and request exists, suggesting a need for a wider coverage of the system. Alternatively, physicians may have struggled with the additional steps and efforts needed to be taken in order to use the iGuide, which may lead them to fill out the request form less carefully. This second potential explanation aligns with a recent qualitative survey among iGuide users, in which the majority of users experienced challenges with the integration of the system [31], and the decision from all participating departments in the MIDAS study to discontinue the use of the iGuide after the trial had concluded.

We found small but statistically significant differences in the percentage of women between the unscored requests and the scored requests included in our study. We do not have supporting evidence that can explain this difference. One hypothesis is that there are more clinical pathways for which no matching guideline exists between the indication and request in requests that are more commonly made for women than for men.

Second, our analysis evaluated each imaging request as a discrete event, without accounting for the cumulative context of individual patient trajectories. This means we could not assess whether a patient underwent multiple inappropriate or appropriate exams over time, nor whether imaging was omitted when it may have been indicated, limiting our ability to comment on potential underuse. We also did not capture the sex or gender identity of the requesting physician, which may be a relevant factor, as prior research suggests that provider gender can influence diagnostic and treatment decisions. A previous study investigating CT appropriateness did, however, not find a difference between male or female physicians requesting imaging [32].

Finally, our analysis was limited to binary sex as recorded in the hospital information system, preventing exploration of disparities experienced by transgender, non-binary, or other gender-diverse individuals. Future work should aim to incorporate more inclusive data collection and explore how patient and provider characteristics intersect to shape diagnostic pathways.

## Conclusion

This study found that women were more likely than men to have inappropriate imaging ordered for them, highlighting a small but consistent sex-based disparity. Addressing these gaps will require further research and more sex- and gender-sensitive approaches in clinical decision-making and guideline development to ensure equitable imaging practices.

## Abbreviations

CDSS: Computerized Decision Support System
CI: Confidence interval
CPOE: Computerized physician order entry
CT: Computed tomography
ESR: European Society of Radiology
MIDAS: Medical Imaging Decision And Support
MR: Magnetic resonance imaging
XR: X-ray
